# Robust Disturbance Estimation-Based Control: Case Study on an Airship Attitude Tracking Problem

**DOI:** 10.3390/e28101069

**Published:** 2026-09-29

**Authors:** Adrian-Mihail Stoica, Valentin Pană, Irina Beatrice Ştefănescu

**Affiliations:** Faculty of Aerospace Engineering, National University of Science and Technology POLITEHNICA of Bucharest, 011061 Bucharest, Romania; valentin.pana@upb.ro (V.P.); irina.stefanescu@upb.ro (I.B.Ş.)

**Keywords:** stochastic modeling with state-dependent noise, *H_∞_* robust control, robust Kalman filtering, wind gust estimation

## Abstract

This paper presents a design methodology for the automatic control system of an airship, accounting for atmospheric disturbances and parametric modeling uncertainties. The derived design model is a linear stochastic system with multiplicative noise incorporating both the airship’s uncertain dynamics and the wind model. First, an $H_{\infty }$ state feedback control law is derived for the stochastic system to ensure robust stability and trajectory tracking performance. Subsequently, a robust Kalman filter is designed to estimate the turbulence model states based on available measurements. It is demonstrated that the optimal gain of this robust filter depends on the solution to a coupled system of specific Riccati and Lyapunov equations. Numerical results indicate improved heading-tracking performance for the considered turbulent simulation when the estimated wind-gust states are incorporated into the feedback law. Under parametric uncertainty, the robust Kalman-type filter also exhibits lower sensitivity of the heading-estimation error than the classical Kalman filter.

## 1. Introduction

The analysis of the effects of atmospheric disturbances on aerospace-vehicle performance is an important step in the evaluation and validation of flight control systems. Two main approaches are adopted in the automatic flight control systems design to mitigate their influence on trajectory tracking. A first class includes passive methods aiming to design the control systems in order to reduce the sensitivity to atmospheric disturbances (see, e.g., [1,2]) and adaptive approaches ([3,4]) based on some a priori knowledge of their properties and bounds. The second category refers to active techniques based on wind velocity measurement or indirect estimation using onboard sensors ([5,6,7]). Among the most widely used methods from the latter category are the Kalman-type algorithms. Kalman filters for unmanned aerial vehicles are presented in [8,9,10], while [11] develops a Kalman filter for estimating the atmospheric disturbances encountered by a large commercial aircraft. The purpose of this paper is to present a design method of the automatic flight control system under modeling uncertainties and in the presence of wind disturbances. Although the application presented concerns the control of an airship, the main ideas in the paper can also be applied to other aerospace applications. The reason the application focuses on an airship is due to its high sensitivity to external disturbances. Despite the long history starting from the middle of the 18th century and of their long period of recession due to the accelerated progress of aircraft during the 20th century, the interest in these aerial vehicles increased over the last decades due to some of the advantages they present including long endurance, low operational costs and hovering capabilities. Meanwhile the area of commercial, scientific and military applications diversified including surveillance missions (see, e.g., [12]).

Among the recent projects involving autonomous airships examples worth mentioning are SASS LITE, Pathfinder and LMH-1 developed in the USA, the autonomous flying solar airship LOTTE, in Germany, KARMA and Flying Whales in France, and the uncrewed airship Kelluu in Finland. Various configurations have been proposed for the automatic control system of airships aiming to achieve stability and tracking performances. In [13], a linear quadratic regulator-based approach is used to design the automatic flight control system of an autonomous airship, while in [14] a mixed $H_{2}/H_{\infty }$ PID (Proportional-Integral-Derivative) controller is derived. A mixed $H_{2}/H_{\infty }$ control law is also derived in [15] using a Markovian stochastic model of the airship’s dynamics. Nonlinear approaches for the airship control based on backstepping, model predictive and sliding mode control may be found for instance, in [16,17] and in [18], respectively. Due to their large volume and low operating speeds a common feature of these aerial vehicles is their vulnerability to atmospheric disturbances ([19,20]). The operational significance of wind-induced lateral-directional motion is also documented in airship occurrence investigations. For example, an American Blimp Corporation A-60+ operating in turbulent and gusty conditions exhibited pronounced rolling and yawing motions while being buffeted by the wind [21]. In another occurrence involving an Airship Industries AD 600, a wind gust during take-off caused the airship to roll to the left, resulting in propeller ground contact and subsequent damage to the envelope [22]. Such events illustrate the practical relevance of lateral-directional response and heading control in the presence of atmospheric disturbances.

This paper proposes a control law that incorporates wind gust estimation, aiming to reduce the sensitivity of reference trajectory tracking performance under windy conditions and uncertainties in the vehicle dynamics model. The Kalman filter is widely used for state estimation in systems subject to additive white noise disturbances. As demonstrated in [23], it minimizes the state estimation error variance, provided the system model parameters are known exactly. However, in real-world applications where these parameters are determined with some degree of uncertainty, filtering performance can be significantly degraded. In numerous studies, the problem of robust Kalman filtering is addressed within a purely deterministic framework, employing models with norm-bounded parametric uncertainties. Recursive state estimators are thus derived, with gains adjusted according to a risk-sensitivity parameter that depends on the difference between the uncertain system and the nominal one (see, e.g., [24]). 

In the present work, a stochastic representation of modeling uncertainties has been adopted. This approach is widely used (see, for example, [25,26] and the related references) and relies on the use of Wiener processes to express parameter uncertainties, which are assumed to follow a normal distribution. The method relies on the 3σ rule which ensures, with a 99.73% probability, that uncertain parameters fall within a range of three standard deviations around their nominal values and its application leads to a stochastic system corrupted with state-dependent (multiplicative) white noises. The control law is derived using an optimal $H_{\infty }$ type procedure developed for this class of stochastic systems. The gust estimation based on the measurements of the vehicle’s states is performed using a robust Kalman-type filter derived in Section 6. The main contributions of the paper are as follows:

- Development of a design model for the lateral-directional dynamics of an airship with parametric uncertainties, represented as a stochastic linear system with multiplicative noise.
- $H_{\infty }$ optimal design of a state feedback control law for linear stochastic systems with multiplicative noise.
- Design of a robust Kalman filter for the considered stochastic class of design models. The optimal filter gain is determined in terms of the solutions to a coupled system of a Riccati and a Lyapunov matrix algebraic equation.
- Illustration and validation of the theoretical results for the automatic flight control system of an airship under atmospheric disturbances and modeling uncertainties.

The paper is organized as follows. Section 2 presents the design models of the airship lateral dynamics, of the wind gusts and the design objectives. The representation of the design model with parametric uncertainties as a stochastic linear system with multiplicative white noise is detailed in Section 3. Further, in Section 4 some known results used in the following developments are briefly reminded. Based on a version of the Bounded Real Lemma for stochastic systems with multiplicative noises, an $H_{\infty }$ state feedback control law is derived in Section 5. The design of a novel robust Kalman-type filter is presented in Section 6 using a characterization of the $H_{2}$-type norm of exponentially stable systems in mean square of stochastic systems with multiplicative noises. In Section 7, numerical results are presented and discussed from the perspectives of robust stability, tracking performance, atmospheric disturbances attenuation and control effort reduction. The paper ends with some concluding remarks in Section 8.

**Notation** **1.** *Throughout the paper the superscript ‘T’ stands for matrix transposition, R denotes the set of scalar real numbers whereas $R^{n}$ denotes the n dimensional Euclidean space and $R^{n\times m}$ is the set of all n×m real matrices. By |v| one denotes the Euclidean norm of the n-dimensional vector v and by $\left|A\right|=\lambda_{max}^{\frac{1}{2}}\left(A^{T}A\right)$ for the real matrix $A\in R^{n\times m}$, with $\lambda_{max}\left(\cdot \right)$ denoting the maximal eigenvalue. The notation P>0 (P≥0), for $P\in R^{n\times n}$ means that P is symmetric and positive definite (positive semi-definite). Similarly, P<0 means that the symmetric matrix P is negative definite. The trace of a matrix Z is denoted by tr(Z). By E[·] one denoted the expectation and by $L^{2}\left(\left[0,\infty \right),R^{m}\right)$, the Lebesgue space of all $R^{m}$ valued functions f(t), t∈[0,∞) with the property that $E\left[\int_{0}^{\infty }{\left|f\left(t\right)\right|}^{2}dt\right]<\infty$.*

## 2. Problem Formulation

The linearized lateral dynamics of the airship, neglecting the influence of the wind disturbances, are approximated by

(1) $$\begin{array}{c} {\dot{x}}_{a}\left(t\right)=A_{a}x_{a}\left(t\right)+B_{a}u_{a}\left(t\right), \end{array}$$

where the state vector $x_{a}$ includes the lateral velocity *v*, the roll rate *p*, the yaw rate *r*, the roll angle ϕ and the heading angle ψ. The components of the control vector $u_{a}$ are the stern rotor thrust denoted by $T_{t}$ and the deflections $\Delta_{1}$ and $\Delta_{2}$ of two aerodynamic surfaces. For the AS500 airship considered in the case study of this paper, the matrices $A_{a}$ and $B_{a}$ of the linearized model corresponding to the trim conditions for straight and level flight at a speed of 6.99 m/s and the altitude h=1000 m are ([13]):

$$\begin{array}{c} \begin{array}{c} A_{a}=\left[\begin{array}{ccccc} -11.38 & -44.45 & 0.86643 & -10.345 & 0\\ -8.9358 & -49.747 & 1.6226 & -11.586 & 0\\ -2.4804 & -9.3868 & -0.050413 & -2.1936 & 0\\ 0 & 1 & -0.052293 & 0 & 0\\ 0 & 0 & 1.0014 & 0 & 0 \end{array}\right],\\ B_{a}=\left[\begin{array}{ccc} -0.11206 & -5.0029 & 5.0029\\ -0.10755 & -3.793 & 3.793\\ -0.032625 & -1.133 & 1.133\\ 0 & 0 & 0\\ 0 & 0 & 0 \end{array}\right]. \end{array} \end{array}$$

The present study focuses on the lateral-directional subsystem, for which the linearized AS500 model is available. Consistently with this modeling scope, the atmospheric disturbance considered below is restricted to the lateral gust component. Extension to longitudinal and vertical gust components would require the corresponding longitudinal or full six-degree-of-freedom airship dynamics and is beyond the scope of the present study.

The lateral velocity *v* of the aircraft is affected by the horizontal speed gust $v_{g}$ approximated as the output of the Dryden filter having the transfer function ([27])

(2) $$\begin{array}{c} H_{v_{g}}\left(s\right)=\sigma_{v}\sqrt{\frac{2L_{v}}{\pi V}}\frac{1+\frac{2\sqrt{3}L_{v}}{V}s}{{\left(1+\frac{2L_{v}}{V}s\right)}^{2}} \end{array}$$

excited with a white noise input signal. The parameters arising in the above transfer function are the scale length $L_{v}$, the speed *V* and the turbulence intensity $\sigma_{v}$. To avoid an excessive complexity of the mathematical models, the yaw turbulence was neglected. Denoting by $\left(A_{v_{g}},B_{v_{g}},C_{v_{g}}\right)$ a realization of the transfer function $H_{v_{g}}$, a state space representation of the Dryden filter dynamics is given by the following Equations

(3) $$\begin{array}{c} \begin{array}{ccc} {\dot{x}}_{g}\left(t\right) & = & A_{v_{g}}x_{g}\left(t\right)+B_{v_{g}}\xi \left(t\right)\\ v_{g}\left(t\right) & = & C_{v_{g}}x_{g}\left(t\right) \end{array} \end{array}$$

where the white noise ξ has zero mean and unit variance.

The regulated output considered in the case study presented in Section 7 is the heading angle ψ. In order to achieve zero steady state errors for piecewise constant values of the commanded heading angle $\psi_{com}$ an integral component is introduced as follows

(4) $$\begin{array}{c} {\dot{x}}_{i}\left(t\right)=\psi_{com}\left(t\right)-\psi \left(t\right). \end{array}$$

Coupling Equations (1), (3) and (4), one obtains the open loop linearized model

(5) $$\begin{array}{c} \dot{x}\left(t\right)=Ax\left(t\right)+B_{1}\psi_{com}\left(t\right)+B_{2}u_{a}\left(t\right)+D\xi \left(t\right) \end{array}$$

in which $x:={\left[\begin{array}{ccc} x_{a}^{T} & x_{g}^{T} & x_{i} \end{array}\right]}^{T}$ and

(6) $$A:=\left[\begin{array}{ccc} A_{a} & B_{v}C_{v_{g}} & 0\\ 0 & A_{v_{g}} & 0\\ -C_{\psi } & 0 & 0 \end{array}\right],\;B_{1}:=\left[\begin{array}{c} 0\\ 0\\ 1 \end{array}\right],\;B_{2}:=\left[\begin{array}{c} B_{a}\\ 0\\ 0 \end{array}\right],\;D:=\left[\begin{array}{c} 0\\ B_{v_{g}}\\ 0 \end{array}\right]$$

with $B_{v}:=A_{a}\left(:,1\right)$ and $C_{\psi }:=\left[\begin{array}{ccccc} 0 & 0 & 0 & 0 & 1 \end{array}\right]$. The above model corresponds to the case in which no modeling uncertainties are considered. In the next section a stochastic model will be developed assuming that some of the parameters are subject to modeling uncertainties.

## 3. Stochastic Representation of the Robust Control and Filtering Design Model

In order to represent the parametric uncertainties of some parameters arising in the model (5), the 3σ-rule will be used as follows: assume that the element (1,1) of the matrix $A_{a}$ from (6) is subject to a ±20% modeling uncertainty. Then, denoting by ${\bar{a}}_{11}=-11.38$ the nominal value of the element (1,1), its uncertain value may be expressed as $a_{11}={\bar{a}}_{11}+\mu_{1}$ where $\mu_{1}$ is a white noise with the variance $\sigma_{\mu_{1}}^{2}$. According to the 3σ-rule, the probability $P\left(|a_{11}-{\bar{a}}_{11}|\leq 3\sigma_{\mu_{1}}\right)\geq 0.9973$ from which one obtains that $\sigma_{\mu_{1}}=0.7587$.

Similarly, we also considered parametric modeling uncertainties of ±10% for the elements (1,2) and (2,2) of $A_{a}$ and for the elements (1,2) and (1,3) of $B_{a}$. Let us notice that the elements (1,2) and (1,3) have the same amplitude but with opposite signs. The same remark is true for the elements (2,2) and (2,3). To conclude, the matrices *A* and $B_{2}$ in (6) may be expressed as

(7) $$\begin{array}{c} A=A_{0}+\sum_{i=1}^{5}\mu_{i}A_{i}\;\;\mathrm{and}\;\;B_{2}=B_{2,0}+\sum_{i=1}^{5}\mu_{i}B_{2,i} \end{array}$$

with the independent white noises $\mu_{i},\;i=1,\ldots ,5$ having the unit variances where

(8) $$\begin{array}{c} \begin{array}{c} A_{0}=A,\;A_{1}=\sigma_{\mu_{1}}\left[\begin{array}{c} \begin{array}{cccc} 1 & 0 & 1 & 0_{1\times 5} \end{array}\\ 0_{7\times 8} \end{array}\right],\;A_{2}=\sigma_{\mu_{2}}\left[\begin{array}{c} \begin{array}{ccc} 0 & 1 & 0_{1\times 6} \end{array}\\ 0_{7\times 8} \end{array}\right],\\ A_{3}=\sigma_{\mu_{3}}\left[\begin{array}{c} 0_{1\times 8}\\ \begin{array}{ccc} 0 & 1 & 0_{1\times 6} \end{array}\\ 0_{6\times 8} \end{array}\right],\;A_{4}=A_{5}=0_{8\times 8},B_{2,0}=B_{2},\;\\ B_{2,1}=B_{2,2}=B_{2,3}=0_{8\times 3},\;B_{2,4}=\sigma_{\mu_{4}}\left[\begin{array}{c} \begin{array}{ccc} 0 & 1 & -1 \end{array}\\ 0_{7\times 3} \end{array}\right],\;B_{2,5}=\sigma_{\mu_{5}}\left[\begin{array}{c} \begin{array}{ccc} 0 & 0 & 0 \end{array}\\ \begin{array}{ccc} 0 & 1 & -1 \end{array}\\ 0_{6\times 3} \end{array}\right] \end{array} \end{array}$$

with $\sigma_{\mu_{1}}=0.7587$, $\sigma_{\mu_{2}}=1.4816$, $\sigma_{\mu_{3}}=1.6582$, $\sigma_{\mu_{4}}=0.1667$ and $\sigma_{\mu_{5}}=0.1264$. Using the above expressions of the matrices *A* and $B_{2}$ and taking into account that the white noises $\mu_{i}\left(t\right),i=1,\ldots ,5$ and ξ(t) can be expressed as derivatives of the Wiener processes $w_{i}\left(t\right),i=1,\ldots ,5$ and β(t), respectively, it results that the open loop system (5) with uncertain parameters may be expressed as

(9) $$\begin{array}{c} dx\left(t\right)=\left(A_{0}x\left(t\right)+B_{1}\psi_{com}\left(t\right)+B_{2,0}u_{2}\left(t\right)\right)dt+\sum_{i=1}^{5}\left(A_{i}x\left(t\right)+B_{2,i}u_{2}\left(t\right)\right)dw_{i}\left(t\right)+Dd\beta \left(t\right), \end{array}$$

representing a stochastic linear system with multiplicative and additive white noise.

## 4. Some Known Useful Results

In this section, some known results used in the following sections will be briefly reminded. Consider the linear stochastic system with multiplicative state-dependent noise

(10) $$\begin{array}{c} \begin{array}{ccc} dx(t) & = & \left(A_{0}x\left(t\right)+Bu\left(t\right)\right)dt+\sum_{i=1}^{r}A_{i}x\left(t\right)dw_{i}\left(t\right)\\ y(t) & = & Cx(t)+Du(t) \end{array} \end{array}$$

where $x\in R^{n}$ denotes the state vector, $u\in R^{m}$ is the input variable and $y\in R^{p}$ denotes its output. The matrices, $A_{i},i=0,\ldots ,r,\;B,\;C$ and *D* are constant and $w_{i},\;i=1,\ldots ,r$ are assumed independent scalar standard Wiener processes on a probability field (Ω,F,P).

**Definition** **1.** *The stochastic system (10) with state-dependent noise is exponentially stable in mean square (ESMS) if there exist β≥1 and α>0 such that ${E[|\Phi \left(t\right)|}^{2}]\leq \beta e^{-\alpha t}$ for all t≥0, where Φ(t) denotes its fundamental (random) matrix.*

The exponential stability in mean square of the stochastic system (10) is characterized by the following result (see, e.g., [28,29]).

**Proposition** **1.** *The stochastic linear system with state-dependent noise (10) is ESMS if and only if there exists a matrix X>0 such that $A_{0}^{T}X+XA_{0}+\sum_{i=1}^{r}A_{i}^{T}XA_{i}<0$.*

If the stochastic system (10) is ESMS, then one can consider the linear and bounded operator

$$\begin{array}{c} T:L^{2}\left(\left[0,\infty \right),R^{m}\right)\to L^{2}\left(\left[0,\infty \right),R^{p}\right) \end{array}$$

defined as

$$\begin{array}{c} \left(Tu\right)\left(t\right)=y_{u}\left(t\right)=Cx_{u}\left(t\right)+Du\left(t\right),\;t\geq 0, \end{array}$$

where $x_{u}$ denotes the solution of the first stochastic differential equation of (10) with null initial condition at t=0. The next result represents a version of the Bounded Real Lemma from the deterministic framework for stochastic systems with state-dependent noises and its proof may be found for instance, in [30,31].

**Lemma** **1.** *If the stochastic system (10) is ESMS, then ∥T∥ < γ for a given γ>0, if and only if $D^{T}D<\gamma^{2}I_{p}$ and if there exists X>0 such that*

(11) $$\begin{array}{c} A_{0}^{T}X+XA_{0}+\sum_{i=1}^{r}A_{i}^{T}XA_{i}+\left(XB+C^{T}D\right){\left(\gamma^{2}I_{p}-D^{T}D\right)}^{-1}\left(B^{T}X+D^{T}C\right)+C^{T}C<0. \end{array}$$

Consider the stochastic linear system with both multiplicative and additive noise

(12) $$\begin{array}{c} \begin{array}{ccc} dx(t) & = & A_{0}x\left(t\right)dt+\sum_{i=1}^{r}A_{i}x\left(t\right)dw_{i}\left(t\right)+D\beta \left(t\right)\\ z(t) & = & Cx(t) \end{array} \end{array}$$

in which $w_{i},i=1,\ldots ,r$ and β are independent Wiener processes. The following result the proof of which may be found for instance, in [32], represents a generalization of the well-known $H_{2}$ -norm definition and expression from the case of systems without state-dependent noises.

**Proposition** **2.** *Assume that the stochastic system (12) denoted by G is ESMS. Then $\lim_{t\to \infty }E\left[|z\left(t\right)|\right]$ with z determined for null initial condition of (12) is well-defined and its $H_{2}$-type norm is by definition, ${∥G∥}_{2}:={\left\{\lim_{t\to \infty }{E[|z\left(t\right)|}^{2}]\right\}}^{\frac{1}{2}}$. Moreover, ${∥G∥}_{2}=tr{\left(C^{T}PC\right)}^{\frac{1}{2}}$ where P≥0 is the controllability Gramian of (12) defined as the unique solution of the Lyapunov-type equation*

$$\begin{array}{c} A_{0}P+PA_{0}^{T}+\sum_{i=1}^{r}A_{i}PA_{i}^{T}+DD^{T}=0. \end{array}$$

The next result useful for the design of the robust Kalman filter in Section 6, gives a monotonicity property of the stabilizing solution to algebraic Riccati equations with respect to their free term (see, e.g., [33]).

**Proposition** **3.** *If X≥0 and $\tilde{X}\geq 0$ are the stabilizing solutions of the Riccati equations:*

$$\begin{array}{c} AX+XA^{T}-XMX+N_{1}=0 \end{array}$$

*and*

$$\begin{array}{c} A\tilde{X}+\tilde{X}A^{T}-\tilde{X}M\tilde{X}+N_{2}=0, \end{array}$$

*respectively (that is A−XM and $A-\tilde{X}M$ are Hurwitz), where M≥0 and $N_{2}\geq N_{1}\geq 0$, then $\tilde{X}\geq X$.*

Finally, the next well-known result is useful to write the nonlinear inequality (11) in an equivalent linear matrix inequality (LMI) form (see, e.g., [31]).

**Proposition** **4.** *The following assertions are equivalent:*

(*i*) *$M=\left[\begin{array}{cc} M_{1} & M_{3}\\ M_{3}^{T} & M_{2} \end{array}\right]<0$;*
(*ii*) *$M_{1}<0$ and $M_{2}-M_{3}^{T}M_{1}^{-1}M_{3}<0$;*
(*iii*) *$M_{2}<0$ and $M_{1}-M_{3}M_{2}^{-1}M_{3}^{T}<0$;*

*where $M_{2}-M_{3}^{T}M_{1}^{-1}M_{3}$ and $M_{1}-M_{3}M_{2}^{-1}M_{3}^{T}$ are the Schur complements of $M_{1}$ and $M_{2}$, respectively.*

## 5. $H_{\infty }$ Type State Feedback Control for the Airship Model with Parametric Uncertainties

We shall present an $H_{\infty }$-type state feedback design procedure for the stochastic system (9) modeling the airship dynamics with parametric uncertainties. To this end we shall introduce the quality output

(13) $$\begin{array}{c} z\left(t\right):=\left[\begin{array}{c} W_{1}x_{i}\left(t\right)\\ W_{2}u\left(t\right) \end{array}\right], \end{array}$$

where $W_{1}\in R$ and $W_{2}\in R^{3\times 3}$ are appropriate positive weights.

The control problem consists in determining the state feedback control u(t)=Fx(t) such that the closed loop system obtained when coupling it to (9) in the absence of the disturbance β(t), namely

(14) $$\begin{array}{c} dx\left(t\right)=\left[\left(A_{0}+B_{2,0}F\right)x\left(t\right)+B_{1}\psi_{com}\left(t\right)\right]dt+\sum_{i=1}^{5}\left(A_{i}+B_{2,i}F\right)x\left(t\right)dw_{i}\left(t\right) \end{array}$$

is ESMS and for a given γ>0, the performance $H_{\infty }$-type index

$$\begin{array}{c} J:=E\left[\int_{0}^{\infty }\left(z^{T}\left(t\right)z\left(t\right)-\gamma^{2}\psi_{com}^{2}\left(t\right)\right)\right]dt<0 \end{array}$$

for all $\psi_{com}\in L^{2}\left(\left[0,\infty \right),R\right)$. Expressing the quality output (13) as z(t)=Mx(t)+Nu(t) with

$$\begin{array}{c} M=\left[\begin{array}{c} W_{1}C_{x_{i}}\\ 0 \end{array}\right]\;\;\mathrm{and}\;\;N=\left[\begin{array}{c} 0\\ W_{2} \end{array}\right] \end{array}$$

where we denoted $C_{x_{i}}:=\left[\begin{array}{cc} 0_{1\times 7} & 1 \end{array}\right]$, based on the Bounded Real Lemma (Lemma 1) presented in Section 4, it follows that the state feedback control problem formulated above is feasible if and only if there exists a matrix X>0 such that

$$\begin{array}{c} \begin{array}{c} {\left(A_{0}+B_{2,0}F\right)}^{T}X+X\left(A_{0}+B_{2,0}F\right)+\sum_{i=1}^{5}{\left(A_{i}+B_{2,i}F\right)}^{T}X\left(A_{i}+B_{2,i}F\right)\\ +\gamma^{-2}XB_{1}B_{1}^{T}X+{\left(M+NF\right)}^{T}{\left(M+NF\right)}^{T}<0. \end{array} \end{array}$$

Based on Schur complements arguments, the above condition is equivalent to the following inequality

(15) $$\begin{array}{c} \left[\begin{array}{cccccc} P_{0}\left(F,X\right) & P_{1}\left(F\right) & \ldots & P_{5}\left(F\right) & XB_{1} & {\left(M+NF\right)}^{T}\\ P_{1}^{T}\left(F\right) & -X^{-1} & \ldots & 0 & 0 & 0\\ \vdots & \vdots & \ldots & \vdots & \vdots & \vdots \\ P_{5}^{T}\left(F\right) & 0 & \ldots & -X^{-1} & 0 & 0\\ B_{1}^{T}X & 0 & \ldots & 0 & -\gamma^{2}I & 0\\ M+NF & 0 & \ldots & 0 & 0 & -I \end{array}\right]<0, \end{array}$$

where we denoted

(16) $$\begin{array}{c} \begin{array}{ccc} P_{0}\left(F,X\right) & := & {\left(A_{0}+B_{2,0}F\right)}^{T}X+X\left(A_{0}+B_{2,0}F\right)\\ P_{i}\left(F\right) & := & {\left(A_{i}+B_{2,i}F\right)}^{T},\;i=1,\ldots ,5. \end{array} \end{array}$$

Inequality (15), which is nonlinear with respect to the variables *F* and X>0, may be transformed into a linear one, by multiplying it to the left and to the right by $diag\left(X^{-1},I,\ldots ,I\right)$ obtaining the equivalent condition:

(17) $$\begin{array}{c} \left[\begin{array}{ccccc} R_{0}\left(Z,Y\right) & R_{1}\left(Z,Y\right) & \ldots & R_{5}\left(Z,Y\right) & YM^{T}+Z^{T}N^{T}\\ R_{1}^{T}\left(Z,Y\right) & -Y & \ldots & 0 & 0\\ \vdots & \vdots & \ddots & \vdots & \vdots \\ R_{5}^{T}\left(Z,Y\right) & 0 & \ldots & -Y & 0\\ MY+NZ & 0 & \ldots & 0 & -I \end{array}\right]<0, \end{array}$$

with, by definition $Z:=FX^{-1}$, $Y:=X^{-1}$,

(18) $$\begin{array}{c} \begin{array}{ccc} R_{0}\left(Z,Y\right) & := & A_{0}Y+YA_{0}^{T}+B_{2,0}Z+Z^{T}B_{2,0}^{T}+\gamma^{-2}B_{1}B_{1}^{T}\\ R_{i}\left(Z,Y\right) & := & YA_{i}^{T}+Z^{T}B_{2,i}^{T},\;i=1,\ldots ,5. \end{array} \end{array}$$

Solving the linear matrix inequalities (LMIs) system (17) with respect to Y>0 and *Z*, one directly obtains the $H_{\infty }$ state feedback gain $F=ZY^{-1}$. The above developments are concluded in the following result.

**Theorem** **1.** *There exists a state feedback gain F for which the stochastic system with multiplicative white noise (14) is ESMS and $E\left[\int_{0}^{\infty }\left(z^{T}\left(t\right)z\left(t\right)-\gamma^{2}\psi_{com}^{2}\left(t\right)\right)\right]dt<0$ for all $\psi_{com}\in L^{2}\left(\left[0,\infty \right),R\right)$ and for a given attenuation level γ>0, if and only if the LMI (17) is feasible with respect to Y>0 and Z, and in this case $F=ZY^{-1}$.*

## 6. Robust Kalman-Type Filtering and Wind Disturbance Estimation

In this section, a novel continuous version of a robust Kalman-type filter is presented, generalizing the result initially derived in [34]. Consider the following ESMS stochastic system with state-dependent noise

(19) $$\begin{array}{c} \begin{array}{ccc} dx(t) & = & A_{0}x\left(t\right)dt+\sum_{i=1}^{r}A_{i}x\left(t\right)dw_{i}\left(t\right)+Dd\beta \left(t\right)\\ dy(t) & = & Cx(t)dt+Hd\eta (t) \end{array} \end{array}$$

where $x\left(t\right)\in R^{n}$ denotes the state, $y\left(t\right)\in R^{p}$ stands for the measured output, β(t),η(t) and $w_{i}\left(t\right),i=1,\ldots ,r$ are zero-mean independent Wiener processes on a given probability space $\left\{\Omega ,F,P\right\}$. It is assumed that *H* is a row full rank matrix. The Kalman type filtering problem treated in this section consists in determining the fixed gain *L* for the filter

(20) $$\begin{array}{c} d\hat{x}\left(t\right)=\left(A_{0}-LC\right)\hat{x}\left(t\right)dt+Ldy\left(t\right), \end{array}$$

such that the matrix $A_{0}-LC$ is Hurwitz and $\lim_{t\to \infty }E\left[|x\left(t\right)-\hat{x}{\left(t\right)|}^{2}\right]$ is minimized.

**Theorem** **2.** *The optimal gain of the Kalman filter (20) is given by*

(21) $$\begin{array}{c} L=XC^{T}{\left(HH^{T}\right)}^{-1} \end{array}$$

*where X denotes the stabilizing solution of the Riccati equation*

(22) $$\begin{array}{c} A_{0}X+XA_{0}^{T}-XC^{T}{\left(HH^{T}\right)}^{-1}CX+\sum_{i=1}^{r}A_{i}YA_{i}^{T}+DD^{T}=0 \end{array}$$

*with Y standing for the unique solution to the Lyapunov matrix equation*

(23) $$\begin{array}{c} A_{0}Y+YA_{0}^{T}+\sum_{i=1}^{r}A_{i}YA_{i}^{T}+DD^{T}=0. \end{array}$$

**Proof.**  Denoting by $e\left(t\right)=x\left(t\right)-\hat{x}\left(t\right)$ the estimation error, one obtains from (19) and (20),

(24) $$\begin{array}{c} \begin{array}{ccc} \left[\begin{array}{c} de(t)\\ dx(t) \end{array}\right] & = & \left[\begin{array}{cc} A_{0}-LC & 0\\ 0 & A_{0} \end{array}\right]\left[\begin{array}{c} e(t)\\ x(t) \end{array}\right]dt+\sum_{i=1}^{r}\left[\begin{array}{cc} 0 & A_{i}\\ 0 & A_{i} \end{array}\right]\left[\begin{array}{c} e(t)\\ x(t) \end{array}\right]dw_{i}\left(t\right)\\ & & +\left[\begin{array}{cc} D & -LH\\ D & 0 \end{array}\right]\left[\begin{array}{c} d\beta (t)\\ d\eta (t) \end{array}\right]. \end{array} \end{array}$$

Based on Proposition 2, it follows that the $H_{2}$-type norm of the above system with the quality output

(25) $$\begin{array}{cc} z(t)= & \left[\begin{array}{cc} I & 0 \end{array}\right]\left[\begin{array}{c} e(t)\\ x(t) \end{array}\right] \end{array}$$

equals trX, where *X* denotes the block (1, 1) of the Gramian

$$\begin{array}{c} \left[\begin{array}{cc} X & Z\\ Z^{T} & Y \end{array}\right] \end{array}$$

of the system (24) which satisfies the Equation

(26) $$\begin{array}{c} \begin{array}{c} \left[\begin{array}{cc} A_{0}-LC & 0\\ 0 & A_{0} \end{array}\right]\left[\begin{array}{cc} X & Z\\ Z^{T} & Y \end{array}\right]+\left[\begin{array}{cc} X & Z\\ Z^{T} & Y \end{array}\right]{\left[\begin{array}{cc} A_{0}-LC & 0\\ 0 & A_{0} \end{array}\right]}^{T}\\ +\sum_{i=1}^{r}\left[\begin{array}{cc} 0 & A_{i}\\ 0 & A_{i} \end{array}\right]\left[\begin{array}{cc} X & Z\\ Z^{T} & Y \end{array}\right]{\left[\begin{array}{cc} 0 & A_{i}\\ 0 & A_{i} \end{array}\right]}^{T}+\left[\begin{array}{cc} D & -LH\\ D & 0 \end{array}\right]{\left[\begin{array}{cc} D & -LH\\ D & 0 \end{array}\right]}^{T}=0. \end{array} \end{array}$$

The block (1, 1) of the above matrix is

$$\begin{array}{c} \left(A_{0}-LC\right)X+X{\left(A_{0}-LC\right)}^{T}+\sum_{i=1}^{r}A_{i}YA_{i}^{T}+DD^{T}+LHH^{T}L^{T}=0, \end{array}$$

which may be rewritten in the equivalent form

(27) $$\begin{array}{c} \begin{array}{c} A_{0}X+XA_{0}^{T}+\sum_{i=1}^{r}A_{i}YA_{i}^{T}+DD^{T}+\left(LH-XC^{T}{H^{†}}^{T}\right)\left(H^{T}L^{T}-H^{†}CX\right)\\ -XC^{T}{\left(HH^{T}\right)}^{-1}CX=0, \end{array} \end{array}$$

where $H^{†}:=H^{T}{\left(HH^{T}\right)}^{-1}$ denotes the pseudoinverse of *H* and where *Y* is the solution of the Lyapunov type matrix Equation (23) representing in fact the block (2, 2) of the matrix equality (26). Using Proposition 3 it follows that the minimal solution X>0 of (27) is obtained if $LH-XC^{T}{H^{†}}^{T}=0$. Thus one obtains the expression (21) from the statement in which case the Equation (27) reduces to (22). Based on the fact that the function tr(X) is increasing with respect to X>0, the conclusion of the statement directly follows. □

**Remark** **1.** *From the statement of the above result it follows that the computation of the optimal Kalman gain L requires solving the coupled system of Riccati and Lyapunov matrix Equations (22) and (23). The Lyapunov Equation (23) depends only on Y and it can be solved by expressing it in the equivalent form*

$$\begin{array}{c} \left(I⨂A_{0}+A_{0}⨂I+\sum_{i=1}^{r}A_{i}⨂A_{i}\right)vec\left(Y\right)=-vec\left(DD^{T}\right) \end{array}$$

*with “⨂” denoting the Kronecker product and where vec(·) stands for the vector obtained by stacking the columns of the matrix (·). The above equality is based on the property that $vec\left(AXB\right)=\left(B^{T}⨂A\right)vec\left(X\right)$ (see, e.g., [35]). It represents a linear algebraic system with respect to vec(Y). Solving this system one obtains Y and after replacing it in (22), the stabilizing solution X directly can be computed directly.*

**Remark** **2.** *It is important to notice a difference between the above robust Kalman filter and the classical one determined in the absence of the multiplicative state dependent white noise terms. In the classical case, a Kalman filter minimizing $\lim_{t\to \infty }E[|x\left(t\right)-\hat{x}\left(t\right)\left|\right]$ may be designed even for unstable plants. For the design of robust Kalman filter presented above, the plant must be ESMS, since if this condition is not fulfilled, due to the instability, the first Equation of (24) would include unbounded noisy components $\sum_{i=1}^{r}A_{i}xdw_{i}$.*

## 7. Numerical Results

The lateral-directional AS500 model was augmented with a second-order Dryden filter describing the lateral atmospheric gust velocity $v_{g}$, together with an integral state associated with the heading-tracking error. The resulting augmented state vector contains the five airship states, namely lateral velocity, roll rate, yaw rate, roll angle and heading angle, the two Dryden-filter states and the integral tracking-error state. The controller was designed for the linearized model at the trim conditions corresponding to a straight and level flight at the forward velocity of $V_{0}$ = 6.99 m/ s and the altitude h=1000 m, consistent with the AS500 configuration considered in [13].

For the simulations reported below, the performance weights were selected as $W_{1}=20$ and $W_{2}=diag\left(12.5,6,6\right)$, and the $H_{\infty }$-type state feedback design was carried out for γ=250. The weights were obtained through a trial-and-error tuning process and should be interpreted as synthesis parameters defining the compromise between the integral heading-tracking error and control effort. In particular, they penalize actuator usage in the performance output, but they do not impose actuator saturation constraints. Solving the LMI (17) with X>0 yields the following state feedback gain *F* used in all the simulations presented in this section

$$\begin{array}{c} F=\left[\begin{array}{cccccccc} -0.5266 & 0.0770 & 2.3459 & -0.0394 & 1.2313 & 0.0707 & 0.0005 & -0.1902\\ -0.3622 & 0.0907 & 9.1251 & -0.0711 & 5.2071 & -0.0856 & -0.0006 & -1.2070\\ 0.3622 & -0.0907 & -9.1251 & 0.0711 & -5.2071 & 0.0856 & 0.0006 & 1.2070 \end{array}\right]. \end{array}$$

The commanded heading is a step of 0.175 rad, corresponding to approximately 10deg, and the simulation interval is 100 s with a time step Δt=0.01. The Dryden model parameters are $L_{v}=266.7$ m, V=6.99 m/s and $\sigma_{v}=2$ m/s. The continuous white-noise processes are approximated numerically by sequences of the form $randn/\sqrt{\Delta t}$, so that the continuous-time noise intensity is preserved under time discretization.

The first simulation isolates the influence of atmospheric turbulence on the controlled heading response. Figure 1 compares the nominal response with the response obtained when the lateral Dryden gust acts on the airship. In the absence of turbulence, the heading angle exhibits a short transient followed by convergence to the commanded value. This behavior is consistent with the integral component introduced in (4), which removes the steady-state tracking error for a constant command.

The atmospheric disturbance produces large deviations of the heading angle around the reference value. This behavior is physically consistent with the comparatively high wind sensitivity of lighter-than-air vehicles, resulting from their low operating velocity and large exposed volume.

The estimation problem is considered next. The measurement vector contains the five lateral-directional airship states and the integral tracking state, whereas the two Dryden-filter states are not directly measured. Measurement noise is introduced through $H=10^{-2}I_{6}$. Two Kalman-type filters were determined; the first one is the classical version derived under the assumption that there are no uncertainties in the airship model, and it has the optimal gain

$$\begin{array}{c} L_{KF}=\left[\begin{array}{cccccc} 4.4169 & 0.8508 & 0.7048 & 0.2350 & 0.1763 & -0.0336\\ 0.8508 & 0.2950 & 0.1737 & 0.0304 & 0.0123 & 0.0074\\ 0.7048 & 0.1737 & 0.1409 & 0.0313 & 0.0160 & 0.0135\\ 0.2350 & 0.0304 & 0.0313 & 0.0150 & 0.0141 & -0.0042\\ 0.1763 & 0.0123 & 0.0160 & 0.0141 & 0.0193 & -0.0011\\ -94.5369 & -24.6304 & -17.4037 & -4.3076 & -2.7480 & -0.5375\\ -24.6280 & -1.7193 & -1.6870 & -1.4478 & -1.3773 & 1.3869\\ -0.0336 & 0.0074 & 0.0135 & -0.0042 & -0.0011 & 0.0294 \end{array}\right]. \end{array}$$

The robust Kalman-type gain was further determined by solving the coupled Lyapunov and Riccati equations derived in Section 6, in which the multiplicative-noise channels associated with the parametric uncertainties are explicitly taken into account. The obtained gain of the robust Kalman filter is:

$$\begin{array}{c} L_{RKF}=\left[\begin{array}{cccccc} 224.5821 & -8.0429 & -1.0084 & 0.0010 & -0.0028 & -0.0062\\ -8.0429 & 36.3344 & -3.6609 & 0.6738 & 0.0284 & -0.0507\\ -1.0084 & -3.6609 & 2.3424 & -0.1153 & 0.4165 & -0.0396\\ 0.0010 & 0.6738 & -0.1153 & 0.9434 & -0.0484 & 0.0040\\ -0.0028 & 0.0284 & 0.4165 & -0.0484 & 0.6690 & -0.4582\\ -5.9153 & -30.2077 & -74.7220 & -17.4458 & -46.8862 & 24.2211\\ -1.8175 & -11.0375 & -29.5165 & -27.3519 & -54.7127 & 62.3285\\ -0.0062 & -0.0507 & -0.0396 & 0.0040 & -0.4582 & 0.8380 \end{array}\right]. \end{array}$$

Figure 2 shows that the robust estimate follows the actual heading over the complete simulation interval, including the time intervals in which the Dryden excitation produces the largest variations. The significance of this result is not restricted to the reconstruction of ψ itself. The filter estimates the complete augmented state, including the unmeasured disturbance states, and can therefore provide the variables required by different feedback control laws. The dashed line represents the time response of the heading angle when the wind estimation is excluded from the control law. 

The sensitivity of the robust estimator to parametric variations was further examined by considering a family of uncertain closed-loop models. The five normalized uncertainty parameters were allowed to vary independently within the interval [−3,3], corresponding to parameter variations within ±3σ, and 200 realizations of the uncertain model were generated. For each stable realization, the same command and stochastic inputs were applied, while the robust filter gain was kept fixed at its nominally designed value. Figure 3 shows a representative subset of the resulting heading estimates together with the nominal response.

These results show that the robust filter is only moderately affected by the considered parameter variations. The uncertainty mainly changes the size of the local deviations, while the overall shape of the estimated response remains close to the nominal one. For most of the simulation interval, the nominal trajectory lies within the family of dispersed responses, indicating that the main dynamic features of the estimate are preserved for the sampled uncertain models.

Since the practical usefulness of an improved disturbance-rejection mechanism also depends on the required control activity, the corresponding actuator demands were evaluated from the estimated state vector according to $u=F\hat{x}$. For the analysis of the actuator response, the commanded control signals were subsequently passed through the first-order actuator dynamics $G_{act}\left(s\right)=1/\left(\tau_{act}s+1\right)$, with time constant $\tau_{act}=0.1$ s. Figure 4 presents the resulting stern-thrust variation together with the two aerodynamic-surface deflections.

The actuator time responses depicted in Figure 4 remain bounded and the actuator dynamics suppresses the highest-frequency content of the commands. The stern-thrust variation is comparatively smooth and remains within a few newtons relative to the trim condition value of 0.2N, whereas the aerodynamic channels exhibit substantially faster and larger excursions because they provide most of the lateral-directional correction required against the turbulent disturbance.

An important remark is required when interpreting Figure 4. The first-order actuator model limits the response bandwidth but does not impose hard position saturation. Accordingly, the plotted signals are unsaturated actuator demands, not constrained physical deflections. The present result should therefore be viewed as a control-effort assessment rather than as a verification of actuator-limit satisfaction. A saturation-aware validation, or a retuning of $W_{2}$ if strict deflection limits are required, represents a natural refinement of the numerical study. Another measure taken to prevent control saturation was the selection of the attenuation level γ. Thus, although a minimal value of γ would be desirable from the standpoint of disturbance attenuation performance, the solution to the $H_{\infty }$ problem in this case would involve higher gains that would promote the occurrence of saturation. 

The main objective of the proposed estimator is robustness with respect to model uncertainty. Its performance was therefore compared with that of a conventional continuous-time Kalman filter. The classical estimator was designed for the nominal closed-loop state matrix $A_{0}+B_{2,0}F$, using the Dryden driving channel as process-noise input with $Q_{KF}=1$ and the same measurement-noise covariance $R_{KF}=HH^{T}$. In contrast with the robust filter derived in Section 6, its design does not explicitly include the five multiplicative-noise channels associated with the uncertain state and control coefficients.

To assess the influence of parametric uncertainty on the estimation performance, the five uncertain parameters were tested at the 1σ, 2σ, and 3σ levels. At each level, all $2^{5}=32$ combinations of positive and negative parameter deviations were considered. Thus, for a given level kσ,k=1,2,3 each normalized uncertainty coefficient was assigned either ±k. Since the standard deviations associated with the uncertain parameters are already included in the corresponding uncertainty matrices, these values represent directly the 1σ, 2σ, and 3σ parameter variations. The nominal case corresponds to zero parametric uncertainty. The same heading command, Dryden disturbance realization and measurement-noise realization were used for the robust and classical filters in all cases.

Figure 5 compares the two estimators for the nominal model and, at each 1σ, 2σ, and 3σ uncertainty level, for the corner combination that produces the largest estimation error among the 32 tested parameter combinations. For the nominal model, the classical Kalman filter gives the smallest estimation error, as expected from its nominal optimality. As the parameter deviations increase, however, its performance deteriorates more rapidly. The robust filter is less affected by the same model variations and maintains a response closer to the actual heading at the 1σ, 2σ, and 3σ levels.

The influence of parametric uncertainty on the estimation accuracy was further evaluated using the Root Mean Square Error (RMSE). For each uncertainty level, the RMSE was computed for all 32 corner combinations, and the largest value was retained. Figure 5 therefore compares the worst-case estimation errors obtained by the two filters within the considered set of parameter variations.

Figure 6 summarizes this behavior in terms of the worst-case RMSE obtained over the 32 parameter combinations at each uncertainty level. The classical Kalman filter provides the best result for the nominal model, but its worst-case estimation error increases significantly with the magnitude of the parameter variations. The robust filter shows a much smaller increase, which confirms its lower sensitivity to the considered modeling uncertainties.

The resulting values are shown in Table 1 which provides a compact confirmation of the behavior observed in Figure 6.

The numerical results show two complementary effects. Firstly, the $H_{\infty }$-type feedback law provides stable heading tracking and attenuates the effect of atmospheric disturbances, at the price of a non-negligible actuator demand during the most energetic gust intervals. Secondly, the robust Kalman-type filter significantly limits the degradation of state-estimation accuracy produced by the considered parametric uncertainty. This latter property becomes increasingly important as the actual plant departs from its nominal configuration and constitutes the principal numerical advantage of the proposed estimator over the classical Kalman solution.

## 8. Conclusions

An optimal control and filtering problem for an airship is analyzed, incorporating wind speed estimation into the control law to improve the vehicle’s reference trajectory tracking performance. Parametric modeling uncertainties are also considered by adopting a stochastic representation with multiplicative white noise for the airship’s automatic flight control system design model. Wind speed estimation was performed using a robust Kalman filter designed for a linear stochastic model with state-dependent noise. The estimated wind speed was subsequently incorporated into a state feedback control law, synthesized using a variant of the Bounded Real Lemma applicable to the class of stochastic systems under consideration. Analysis of the numerical results indicates improvements in both tracking performance and robustness compared to scenarios where the control law does not include wind disturbance estimation or where a classical Kalman filter is used. 

## Figures and Tables

**Figure 1:**
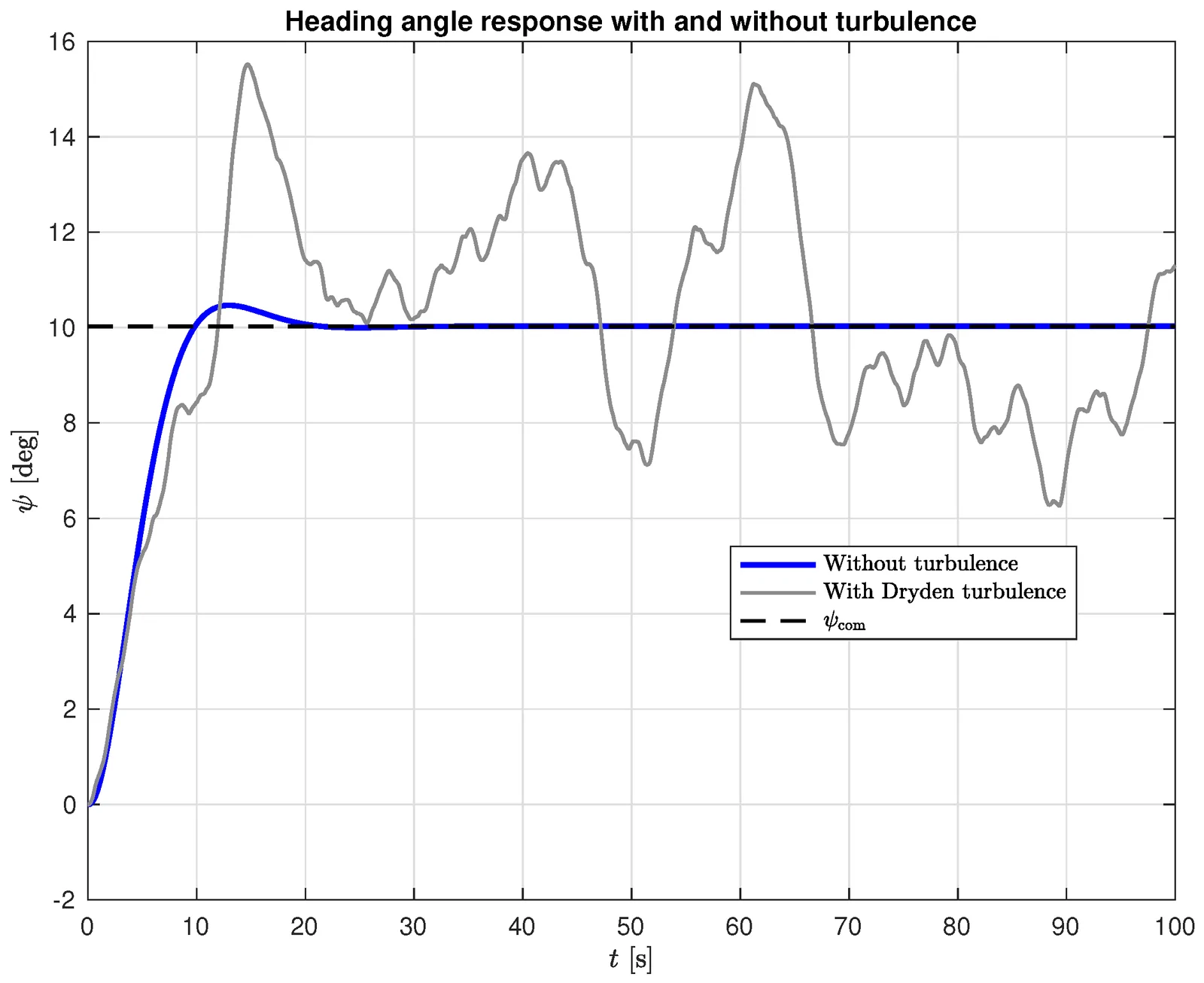
Heading-angle response to a 0.175 rad (approximately 10deg) command in the absence and in the presence of lateral Dryden turbulence.

**Figure 2:**
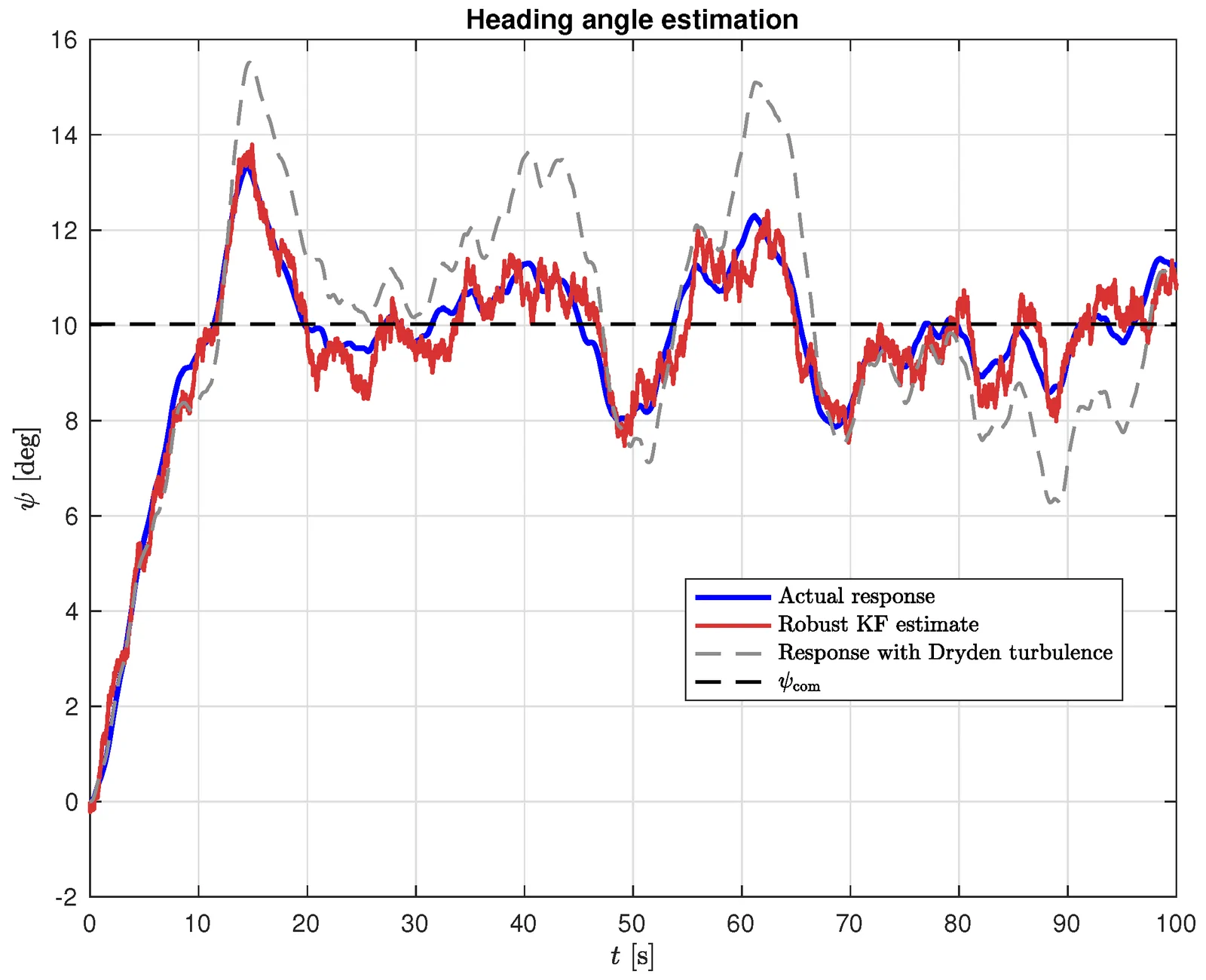
Actual heading response and robust Kalman-type estimate in the presence of atmospheric turbulence.

**Figure 3:**
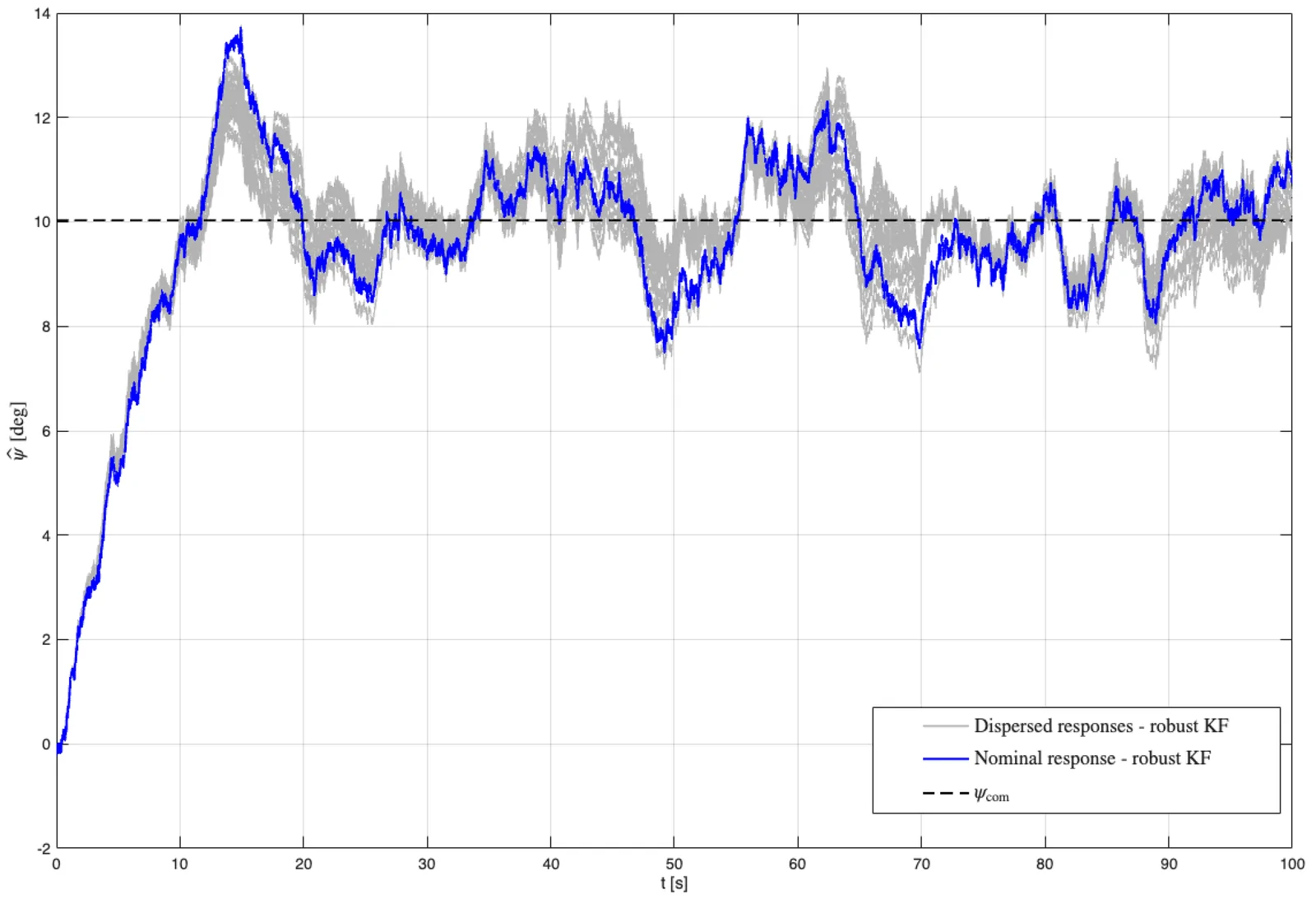
Selected dispersed responses—robust KF.

**Figure 4:**
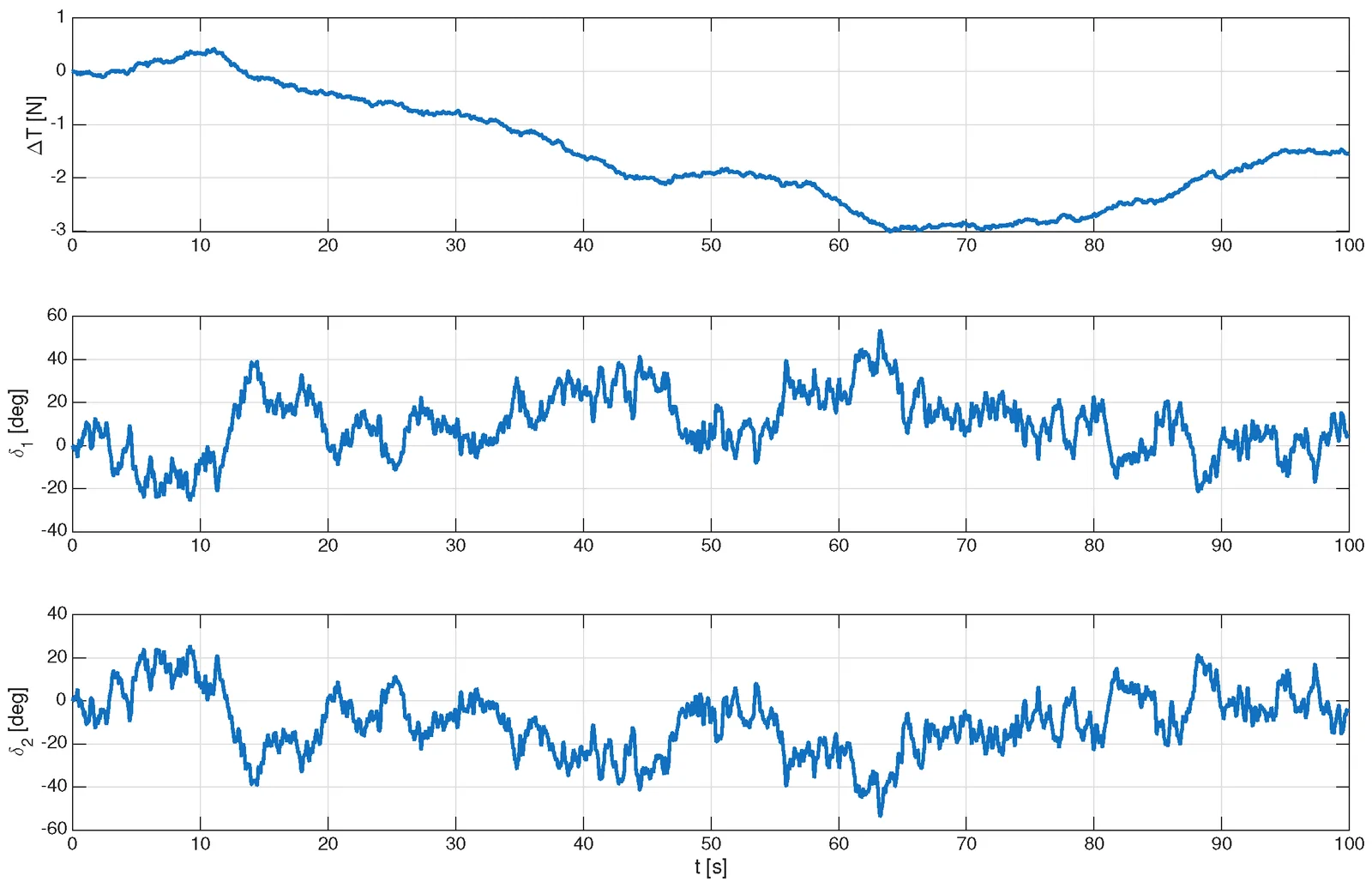
Actuator responses computed from the estimated states and filtered through the first-order actuator dynamics.

**Figure 5:**
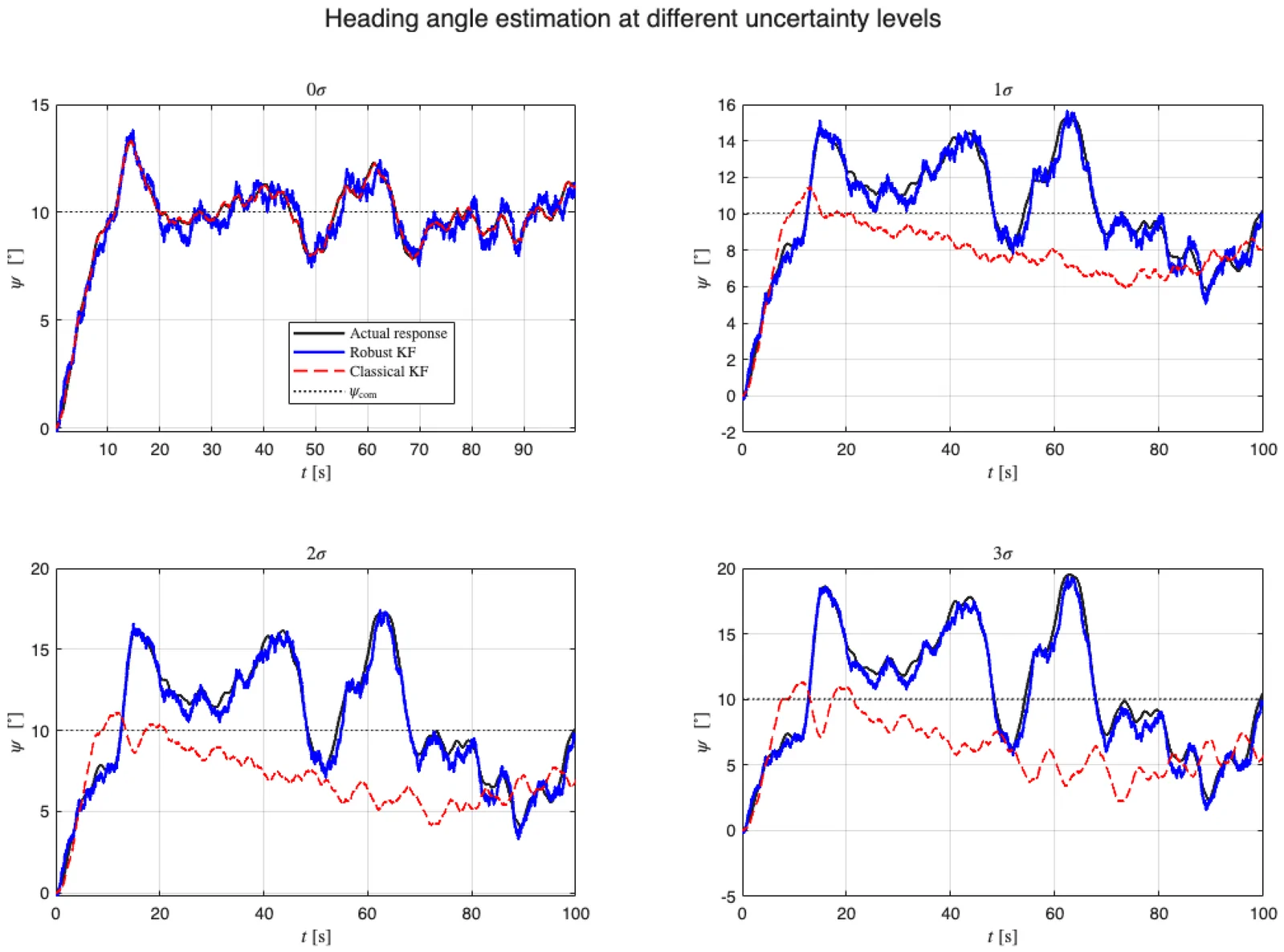
Comparison of robust and classical Kalman-filter heading estimates for increasing levels of parametric uncertainty.

**Figure 6:**
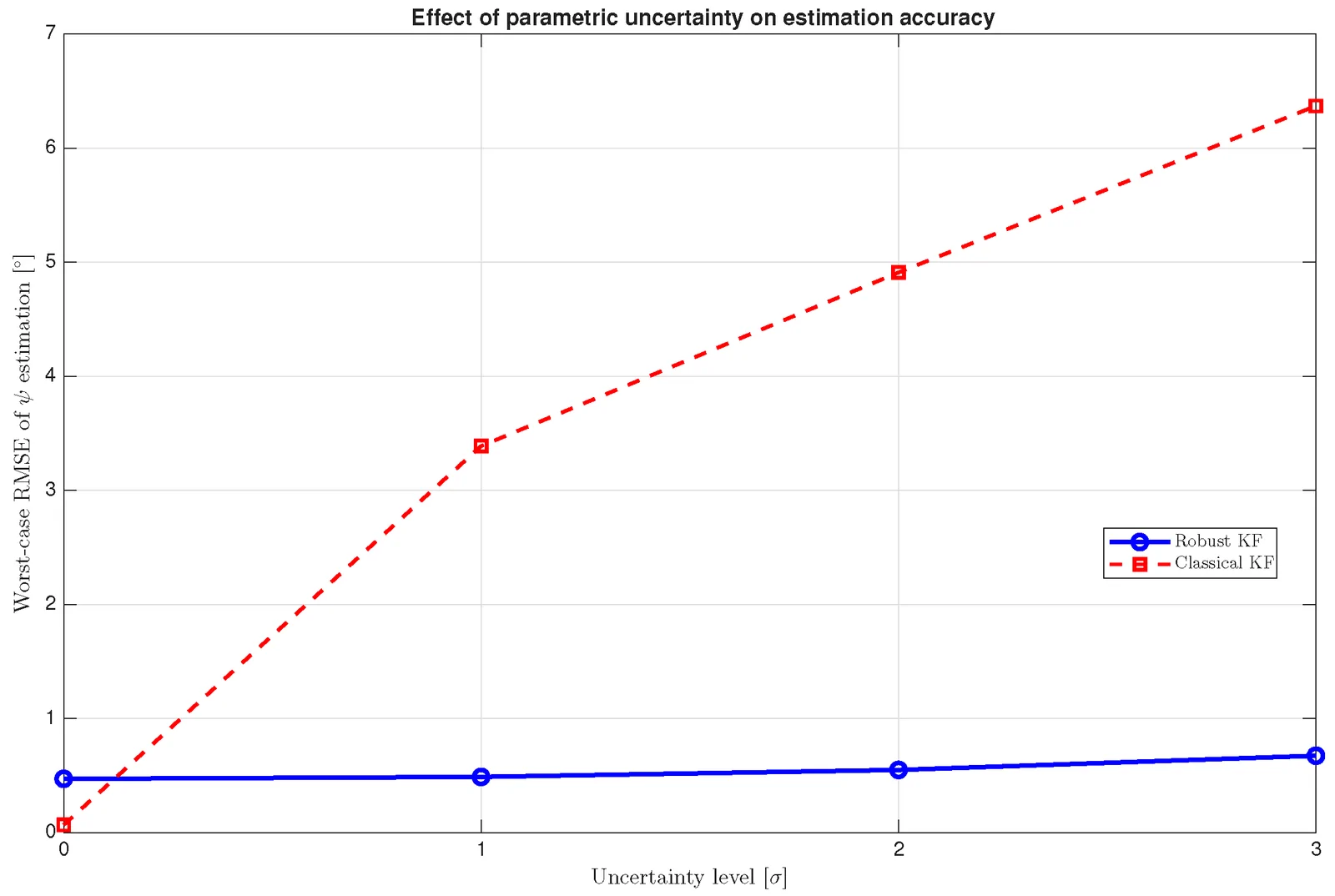
Root-mean-square heading estimation error of the robust and classical Kalman filters versus parametric uncertainty level.

**Table 1:** Summary of worst-case RMSE.

Uncertainty Level	Classical KF [deg]	Robust KF [deg]
0σ	0.0684	0.4719
1σ	3.3887	0.4890
2σ	4.9103	0.5502
3σ	6.3702	0.6747

## Data Availability

The original contributions presented in this study are included in the article. Further inquiries can be directed to the corresponding author.
